# Enteric Infections Circulating during Hajj Seasons, 2011–2013

**DOI:** 10.3201/eid2310.161642

**Published:** 2017-10

**Authors:** Moataz Abd El Ghany, Mona Alsomali, Malak Almasri, Eriko Padron Regalado, Raeece Naeem, AbdulHafeez Tukestani, Abdullah Asiri, Grant A. Hill-Cawthorne, Arnab Pain, Ziad A. Memish

**Affiliations:** King Abdullah University of Science and Technology, Thuwal, Saudi Arabia (M. Abd El Ghany, M. Alsomali, E. Padron Regalado, R. Naeem, A. Pain);; University of Sydney, Australia (M. Abd El Ghany, G.A. Hill-Cawthorne);; Saudi Arabia Ministry of Health, Riyadh, Saudi Arabia (M. Almasri, A. Tukestani, A. Asiri, Z.A. Memish);; Hokkaido University, Sapporo, Japan (A. Pain);; Alfaisal University, Riyadh (Z.A. Memish);; Emory University, Atlanta, Georgia, USA (Z.A. Memish)

**Keywords:** Hajj, mass gathering, enteric infections, diarrhea, antimicrobial resistance, viruses, bacteria, Saudi Arabia

## Abstract

Hajj, the annual Muslim pilgrimage to Mecca, Saudi Arabia, is a unique mass gathering event that raises public health concerns in the host country and globally. Although gastroenteritis and diarrhea are common among Hajj pilgrims, the microbial etiologies of these infections are unknown. We collected 544 fecal samples from pilgrims with medically attended diarrheal illness from 40 countries during the 2011–2013 Hajj seasons and screened the samples for 16 pathogens commonly associated with diarrheal infections. Bacteria were the main agents detected, in 82.9% of the 228 positive samples, followed by viral (6.1%) and parasitic (5.3%) agents. *Salmonella* spp., *Shigella*/enteroinvasive *Escherichia coli*, and enterotoxigenic *E. coli* were the main pathogens associated with severe symptoms. We identified genes associated with resistance to third-generation cephalosporins ≈40% of *Salmonella*- and *E. coli*–positive samples. Hajj-associated foodborne infections pose a major public health risk through the emergence and transmission of antimicrobial drug–resistant bacteria.

## Abstract 2

Foodborne-associated bacteria with increased incidence of antimicrobial drug resistance were the most common cause.

Hajj, the annual pilgrimage by Muslims to Mecca, Saudi Arabia, is a unique mass gathering event in terms of scale (i.e., the number of pilgrims), diversity of the pilgrims, nature of the activities performed, and regularity. Approximately 2 million pilgrims from 185 countries, in addition to hundreds of thousands of residents of Saudi Arabia, travel to holy sites in Mecca each year (*1*). This enormously diverse population (in terms of ethnic origin, socioeconomic status, sex, age, and health status) comes together to perform the same activities within a relatively short period over a limited area of land (*2*), which allows for the mixing of infectious agents and susceptible populations (*3*). Mass gatherings such as Hajj therefore increase the potential for the emergence and dissemination of infections and raises public health concerns in Saudi Arabia and globally (*4*). Hajj-associated communicable public health hazards mainly involve the transmission of respiratory infections, foodborne diseases, bloodborne diseases, and zoonotic infections (*4*).

Globally, diarrheal infections remain the leading cause of mortality in children <5 years of age and contribute to ≈10% of child deaths each year (*5*–*7*). In addition, traveler’s diarrhea is still the most common illness observed in travelers returning from regions where diarrheal diseases are endemic (*8*,*9*). The main etiologic agents detected are consistently bacteria (*Escherichia coli*, *Salmonella* spp., *Shigella* spp., and *Campylobacter* spp.); viruses (rotavirus, norovirus, and adenovirus); and parasites (*Cryptosporidium* spp., *Giardia lamblia*, and *Entamoeba histolytica*) (*10*,*11*).

Despite substantial advances in food and water hygiene in many countries, mass gathering events still represent the perfect environments for the transmission of enteric infections (*12*,*13*). Diarrheal infections and foodborne diseases are commonly associated with the Hajj pilgrimage (*14*). Although diarrheal infections and other enteric infections are one of the most common complaints among pilgrims, little information is available regarding incidence, etiologic agents, and the abundance of antimicrobial drug–resistant strains. Published reports have mainly been based on analyses of hospital admission data that lack full characterization of the nature of the infections (*15*–*17*). Moreover, estimates of the incidence of Hajj-associated gastrointestinal disease based on hospital admission data can vary considerably (*14*). Recently, a few studies have shown an increase in the carriage rates of enteric pathogens that include *Tropheryma whipplei* (*18*), multidrug-resistant nontyphoidal *Salmonella* (*19*), and carbapenemase-producing *E. coli* (*20*) among pilgrims from France returning from Hajj. These findings, coupled with the growing threat of drug-resistant microorganisms (*21*), increase the risks associated with the Hajj pilgrimage and fuels the emergence and dissemination of drug-resistant enteric pathogens.

We conducted a large-scale study to catalog the circulating enteric pathogen population in Hajj pilgrims with diarrheal symptoms. We report on the use of molecular and antigenic approaches to characterize the etiologic agents associated with enteric infections in pilgrims who sought medical treatment while performing Hajj during the 2011–2013 seasons.

## Materials and Methods

### Ethics Statement

The samples were originally collected for diagnostic purposes; therefore, collection was not experimental in nature. The Ministry of Health of Saudi Arabia anonymized all identifiable information, and only deidentified records and samples were available to the researchers. The King Fahad Medical City Institutional review board approved the study protocol (approval no. 11–157, dated October 4, 2011). The Institutional Biosafety and Ethics Committee of King Abdullah University of Science and Technology also approved the study in 2013.

### Study Design

We conducted the study for 3 successive Hajj seasons, starting in 2011. Fecal samples from pilgrims having medically attended diarrhea while performing Hajj were collected. Healthcare facilities distributed along the Hajj sites were enrolled in the study.

We included patients with symptoms who were seeking medical care for diarrhea or who were admitted to hospitals or primary care centers established in the holy sites during the 7–10 day Hajj period. We defined diarrhea as the occurrence of >3 unformed stools in a 24-hour period or passing stool more frequently than normal for the patient, accompanied with >1 other gastrointestinal symptom (abdominal pain/cramps, vomiting, or bloody or mucoid stools). Patients who had unformed stool with visible blood were defined as having cases of dysentery. Patients with increased body temperature were categorized as having either mild (>37.5°C and <39°C) or severe (>39°C) fever.

We categorized the patients into 2 groups according to degree of symptom severity. We defined severe diarrhea as >6 unformed stools per day; diarrhea requiring hospitalization; or diarrhea accompanied by fever, dehydration, or bloody or mucoid stools. We classified patients with diarrhea not fulfilling the criteria for severe symptoms as having mild cases. We screened all the samples molecularly, antigenically, or both for a panel of 16 infectious agents commonly associated with diarrheal infection.

### Antigenic Detection of Viral and Parasitic Pathogens

We used qualitative enzyme immunoassays for the initial detection of viral agents in the fecal samples according to manufacturers’ instructions. We used the IDEIA Norovirus test (Oxoid, Basingstoke, UK) to detect norovirus genogroups 1 and 2 and ProSpecT tests (Oxoid) to detect of group A rotaviruses, adenoviruses, and astroviruses. For parasitic agents, we used the *Giardia*/*Cryptosporidium* Quik Chek test (TechLab, Blacksburg VA, USA) for the detection and differentiation of *Cryptosporidium* oocyst antigen and *Giardia* cyst antigen.

### Isolation of DNA Using QIAsymphony Platform

We used the QIAsymphony SP (QIAGEN, Hilden, Germany), an automated high-throughput platform, for the isolation and purification of total DNA from the collected fecal samples. We used the QIAsymphony DNA 800 complex kit (QIAGEN) to extract DNA from 800 μL of pretreated diluted samples according to the manufacturer’s instructions.

### Molecular Characterization of Bacterial Species

We used 3 previously established multiplex PCR assays (M1, 2, and 3) in parallel to detect the bacterial pathogens commonly associated with diarrheal infections (*22*). The M1 multiplex PCR used primers targeting genes *eae* and *bfpA* (enteropathogenic *E. coli*), *aggR* (enteroaggregative *E. coli*) and Vero cytotoxin (enterohemorrhagic *E. coli*). The M2 multiplex PCR used primers targeting the genes *elt* and *st* (enterotoxigenic *E. coli* [ETEC]), *daaE* (diffusely adherent *E. coli*), and *virF* and *ipaH* (*Shigella* spp./enteroinvasive *E. coli* [EIEC]). The M3 multiplex PCR used primers targeting the *hipO* gene (*Campylobacter jejuni*), internal transcribed spacer region (*Salmonella* spp.), *Yersinia* stable toxin gene (*Yersinia enterocolitica*) and *rtxA* gene (*Vibrio cholerae*). Primer details and the expected PCR fragment sizes are provided (Technical Appendix Table 1). In summary, we mixed 200–400 ng of the extracted total DNA, 1–10 μmol/L of each of the primer pairs, and GoTaq Green Master Mix (Promega, Madison, WI, USA) in a PCR total reaction volume of 25 μL to amplify the target genes. We ran PCR products on a 1.5% agarose electrophoresis gel at 120 volts for 2 hours and identified fragment sizes against positive controls by using the GelPilot 1kb Plus ladder (QIAGEN).

**Table 1:** Demographic characteristics of persons who acquired enteric infections during their travel for Hajj, 2011–2013

Characteristic	Year			Total	Statistical analyses*	
	2011	2012	2013		χ^2^	p value
No. patients	118	297	129	544		
Median patient age, y (quartile deviation)	40 (+12.25)	40 (+13.25)	40.5 (+14.0)	40.17 (+13.17)		
No. countries of origin represented	20	30	20	40		
Sex, no. (%)						
F	32 (27.12)	84 (28.28)	30 (23.26)	146 (26.84)	1.07	0.59
M	86 (72.12)	213 (71.72)	98 (75.97)	397 (72.98)		

*Comparison between Hajj seasons.

### Molecular Characterization of Viral Agents

We used the QIAamp Viral RNA Mini Kit (QIAGEN) to extract viral RNA from antigenically positive samples for rotavirus, norovirus, and astrovirus according to the manufacturer’s instruction. We performed reverse transcription by using the SuperScript III First-Strand Synthesis System (Life Technologies, Carlsbad, CA, USA) and PCR amplification by using Platinum Taq DNA Polymerase High Fidelity (Thermo Fisher Scientific, Waltham, MA, USA) and previously described primers for the detection of rotavirus (*23*), norovirus (*24*), and astrovirus (*25*). Primer details and expected PCR fragment size are provided (Technical Appendix Table 2). We purified PCR products by using the MinElute Gel Extraction kit (QIAGEN); sequencing was performed on an ABI 3730xl (Thermo Fisher Scientific) at the Bioscience Core Laboratory at King Abdullah University of Science and Technology. We used BioEdit Sequence Alignment Editor 7.2.6.1 (http://www.mbio.ncsu.edu/bioedit/page2.html) to trim and align bidirectional sequence reads and used the consensus sequences to identify the viral genotype. We identified rotavirus genotypes by using RotaC version 2.0 software (*26*) and noroviruses by using genotyping tool version 1.0 (*27*). We used previously described phylogenetic analyses to identify astrovirus genotypes (*28*).

**Table 2:** Clinical characteristics of persons who acquired enteric infections during their travel for Hajj, 2011–2013

Characteristic	Year			Total	Statistical analyses*	
	2011	2012	2013		χ^2^	p value
Hospitalization, no. (%)						
Outpatient	94 (79.66)	263 (88.55)	116 (89.92)	473 (86.95)	7.54	0.02
Inpatient	24 (20.34)	33 (11.11)	13 (10.08)	70 (12.87)		
Not defined	0	1 (0.34)	0	1 (0.18)		
Stool consistency, no. (%)						
Unformed†	57 (48.31)	187 (62.96)	76 (58.91)	320 (58.82)	9.19	0.01
Watery‡	60 (50.85)	101 (34.01)	48 (37.12)	209 (38.42)		
Not defined	1 (0.85)	9 (3.03)	5 (3.88)	15 (2.76)		
Abdominal pain/cramp, no. (%)						
Yes	106 (89.83)	282 (94.95)	103 (79.84)	491 (90.26)		
No	0	0	0	0		
Not defined	12 (10.17)	15 (5.05)	26 (20.16)	53 (9.74)		
Bowel movements/d, no. (%)						
<3	9 (7.63)	5 (1.68)	3 (2.33)	17 (3.13)	11.21	0.02
3–5	78 (66.1)	212 (71.38)	76 (58.91)	366 (67.28)		
>5	19 (16.1)	65 (21.89)	24 (18.6)	108 (19.85)		
Not defined	12 (10.17)	15 (5.05)	26 (20.16)	53 (9.74)		
Duration of diarrhea, d, no. (%)						
<2	61 (51.69)	140 (47.14)	67 (51.94)	268 (49.26)	2.99	0.56
3–5	42 (35.59)	119 (40.07)	42 (32.56)	203 (37.32)		
>5	12 (10.17)	24 (8.08)	8 (6.2)	44 (8.09)		
Not defined	3 (2.54)	14 (4.71)	12 (9.3)	29 (5.33)		
Presence of mucus, no. (%)						
Yes	38 (32.2)	165 (55.56)	41 (31.78)	244 (44.85)	32.08	<0.001
No	80 (67.8)	126 (42.42)	86 (66.67)	292 (53.68)		
Not defined	0	6 (2.02)	2 (1.55)	8 (1.47)		
Presence of blood, no. (%)						
Yes	14 (11.86)	26 (8.75)	11 (8.53)	51 (9.38)	0.98	0.61
No	104 (88.14)	265 (89.23)	116 (89.92)	485 (89.15)		
Not defined	0	6 (2.02)	2 (1.55)	8 (1.47)		
Vomiting, no. (%)						
Yes	33 (27.97)	80 (26.94)	12 (9.3)	125 (22.98)	17.92	<0.001
No	85 (72.03)	211 (71.04)	115 (89.15)	411 (75.55)		
Not defined	0	6 (2.02)	2 (1.55)	8 (1.47)		
Fever, no. (%)§						
No fever	87 (73.73)	167 (56.23)	103 (79.84)	357 (65.63)	26.90	<0.001
Moderate	23 (19.49)	72 (24.24)	25 (19.34)	120 (22.06)		
Severe	3 (2.54)	32 (10.77)	1 (0.78)	36 (6.62)		
Not defined	5 (4.24)	26 (8.75)	0	31 (5.7)		
Dehydration, no. (%)¶						
Yes	42 (35.59)	53 (17.85)	39 (30.23)	134 (24.63)	16.89	<0.001
No	76 (64.41)	238 (80.13)	85 (65.89)	399 (73.35)		
Not defined	0	6 (2.02)	5 (3.88)	11 (2.02)		

*Comparison between Hajj seasons. †Bristol 6. ‡Bristol 7.  §Moderate fever defined as >37.5°C and <39.0°C; severe fever defined as >39.0°C. ¶Dehydration defined as >2 of the following signs or symptoms: thirst, dry mouth, weakness/light headedness, and darkening of the urine/decrease in urination.

### Molecular Characterization of β-Lactamase Genes

We further screened the samples positive for 1 of the *Enterobacteriaceae* species for the detection of β-lactamase genes (*bla*_CTX-M-15_, *bla*_IMP_, *bla*_KPC_, *bla*_NDM_, *bla*_OXA-48_, and *bla*_VIM_) as previously described (*29*). The list of primers used in the detection of β-lactamase genes and the expected PCR fragment sizes are provided (Technical Appendix Table 3).

**Table 3:** Characteristics of etiologic agents associated with enteric infections among persons infected during their travel for Hajj, 2011–2013*

Characteristic	Year			Total
	2011	2012	2013	
No. screened samples	118	297	129	544
Samples positive for agent, no. (%)	51 (43.22)	120 (40.40)	57 (44.19)	228 (41.91)
Bacterial agents, no. (%)	41 (34.75)	96 (32.32)	52 (40.31)	189 (34.74)
* Salmonella*	13 (11.02)	25 (8.42)	24 (18.6)	62 (11.4)
*Shigella*/EIEC	5 (4.24)	28 (9.43)	8 (6.2)	41 (7.54)
ETEC	12 (10.17)	29 (9.76)	7 (5.43)	48 (8.82)
EPEC	3 (2.54)	5 (1.68)	8 (6.2)	16 (2.94)
EHEC	2 (1.69)	2 (0.67)	0	4 (0.74)
DAEC	3 (2.54)	1 (0.34)	3 (2.33)	7 (1.29)
EAEC	3 (2.54)	2 (0.67)	2 (1.55)	7 (1.29)
* Yersinia enterocolitica*	0	4 (1.35)	0	4 (0.74)
Viral agents, no. (%)	6 (5.08)	7 (2.36)	1 (0.78)	14 (2.57)
Astrovirus	0	2 (0.67)	1 (0.78)	3 (0.55)
Norovirus	2 (1.69)	2 (0.67)	0	4 (0.74)
Rotavirus	4 (3.39)	2 (0.67)	0	6 (1.1)
Adenovirus	0	1 (0.34)	0	1 (0.18)
Parasitic agents, no. (%)	3 (2.54)	8 (2.69)	1 (0.78)	12 (2.21)
* Giardia*	3 (2.54)	6 (2.02)	1 (0.78)	10 (1.84)
* Cryptosporidium*	0	2 (0.67)	0	2 (0.37)
Mixed infectious agents, no. (%)	1 (0.85)	9 (3.03)	3 (2.33)	13 (2.39)
Bacteria and virus	0	5 (1.68)†	1 (0.78)‡	6 (1.1)
Bacteria and parasite	1 (0.85)§	4 (1.35)¶	1 (0.78)#	6 (1.1)
Bacteria, virus, and parasite	0	0	1 (0.78)**	1 (0.18)

*EAEC, enteroaggregative *Escherichia coli*; EHEC, enterohemorrhagic *E. coli*; EIEC, enteroinvasive *E. coli*; EPEC, enteropathogenic *E. coli*; ETEC, enterotoxigenic *E. coli*; DAEC, diffusely adherent *E. coli*. †*Salmonella* and rotavirus G1P[8], *Shigella*/EIEC and astrovirus HAstV2, ETEC and astrovirus HAstV2, *Salmonella* and adenovirus, and EPEC and rotavirus G1P[8]. ‡EPEC and adenovirus. §*Salmonella* and *Giardia*. ¶EPEC and *Giardia*, EAEC and *Giardia*, ETEC and *Giardia,* and *Salmonella* and *Giardia*. #EPEC and *Cryptosporidium*. **EPEC, adenovirus, and *Giardia*.

### Statistical Analysis

We evaluated the differences between the sets of the categorical data by using the Pearson χ^2^ test. We defined statistical significance as p<0.05.

## Results

### Demographic and Clinical Features of the Patients

During 3 consecutive Hajj seasons (2011–2013), we collected 544 fecal samples from pilgrims who had diarrhea while performing Hajj and who sought treatment at healthcare facilities (Tables 1, 2). These patients originated from 40 countries on 5 continents (Technical Appendix Table 4). Most patients (434, 79.8%) originated from 7 countries: Saudi Arabia (24.82%, n = 135), Nigeria (15.07%, n = 82), Egypt (12.87%, n = 70), Bangladesh (8.09%, n = 44), Pakistan (6.43%, n = 35), Yemen (6.25%, n = 34), and India (6.25%, n = 34). Median (+quartile deviation) patient age was 40.17 (+13.17) years. By Hajj season, median age was 40 (+12.25) years in 2011, 40 (+13.25) in 2012, and 40.5 (+14) years in 2013 (Table 1). Most patients were men (72.98%, n = 397); women represented 27.12% of patients in 2011, 28.28% in 2012, and 23.26% in 2013 (Table 1). 

**Table 4:** Relationship between severity of diarrheal disease and identified etiologic agents among persons who acquired enteric infections during their travel for Hajj, 2011–2013*

Category	Severity of diarrheal disease			Statistical analyses	
	Severe	Mild		χ^2^	p value
Total no. cases	412	132			
Positive for etiologic agent, no. (%)	185 (44.9)	43 (32.58)		6.24†	0.01
Bacterial agents, no. (%)	153 (37.14)	36 (27.27)		4.29‡	0.04
* Salmonella*	45 (10.92)	17 (12.88)		4.19§	0.04
*Shigella*/EIEC	35 (8.5)	6 (4.55)			
ETEC	43 (10.44)	5 (3.79)		5.49¶	0.019
EPEC	13 (3.16)	3 (2.27)			
EHEC	3 (0.73)	1 (0.76)			
DAEC	7 (1.7)	0			
EAEC	5 (1.21)	2 (1.52)			
* Yersinia enterocolitica*	2 (0.49)	2 (1.52)			
Viral agents, no. (%)	13 (3.16)	1 (0.76)		2.29	0.13
Astrovirus	2 (0.49)	1 (0.76)			
Norovirus	4 (0.97)	0			
Rotavirus	6 (1.46)	0			
Adenovirus	1 (0.24)	0			
Parasitic agents, no. (%)	10 (2.43)	2 (1.52)		0.39	0.53
* Giardia*	8 (1.94)	2 (1.52)			
* Cryptosporidium*	2 (0.49)	0			
Mixed infectious agents, no. (%)	9 (2.18)	4 (3.03)		0.31	0.58
Bacteria and virus	4 (0.97)	2 (1.52)			
Bacteria and parasite	5 (1.21)	1 (0.76)			
Bacteria, virus, and parasite	0	1 (0.76)			

*EAEC, enteroaggregative *Escherichia coli*; EHEC, enterohemorrhagic *E. coli*; EIEC, enteroinvasive *E. coli*; EPEC, enteropathogenic *E. coli*; ETEC, enterotoxigenic *E. coli*; DAEC, diffusely adherent *E. coli*; OR, odds ratio. †Compared with total number of cases. OR 1.69, p=0.01. ‡Compared with total number of cases. OR 1.58, p=0.04.  §Compared with total number of positive bacteria. OR 0.47, p=0.04.  ¶Compared with total number of cases. OR 2.96, p=0.02.

We summarized the distribution of the clinical features among the patients during the 3 Hajj seasons (Table 2). Most patients were seen as outpatients (86.95%, n = 473), and the most frequently reported symptoms were abdominal pain/cramp (90.26%, n = 491), presence of mucus in the stool (44.85%, n = 244), watery diarrhea (38.42%, n = 209), dehydration (24.63%, n = 134), vomiting (22.98%, n = 125), and moderate fever (22.06%, n = 120). Less common symptoms were bloody stool (9.38%, n = 51) and severe fever (6.62%, n = 36). We observed significant differences in the frequencies of these symptoms across the 3 Hajj seasons (Table 2).

### Characterization of Bacterial Pathogens

We screened the 544 fecal samples collected from the patients during the 2011–2013 Hajj seasons for 16 infectious agents, including bacteria, viruses, and parasites commonly associated with diarrheal infections. We calculated the number of the samples tested and the number and percentage of the positive samples from each season (Table 3). We detected >1 of the pathogens screened for in 41.91% (n = 228) of the samples. We observed no significant difference between the numbers of positive samples during the 3 seasons (χ^2^ = 0.63; p = 0.73). The percentages of positive samples detected were 43.22% (n = 51) for 2011, 40.40% (n = 120) for 2012, and 44.19% (n = 57) for 2013. Bacterial pathogens were the predominant infectious agents detected for the 3 Hajj seasons and the agents identified in 34.74% (n = 189) of the total samples, followed by viral (2.57%, n = 14) and parasitic (2.21%, n = 12) agents. Thirteen patients (representing 2.39% of the total samples) had samples testing positive for >1 pathogen. We observed no significant difference in the distribution of infectious agents across the 3 seasons (χ^2^ = 8.84; p = 0.18).

We calculated the distribution of patients by age group and the enteric pathogens identified (Figure, panels A, B). The highest proportion of patients having diarrhea of known etiology, compared with unknown, was the <20-year-old age group (odds ratio [OR] 2.46; p = 0.0002). Conversely, the highest proportion of patients having diarrhea of unknown etiology compared with known was the 40–60 years age group (OR 0.52; p = 0.0004). For most of the age groups, bacteria were the main cause of diarrhea in patients, with no significant difference detected across the 3 Hajj seasons (χ^2^ = 8.59; p = 0.2).

**Figure:**
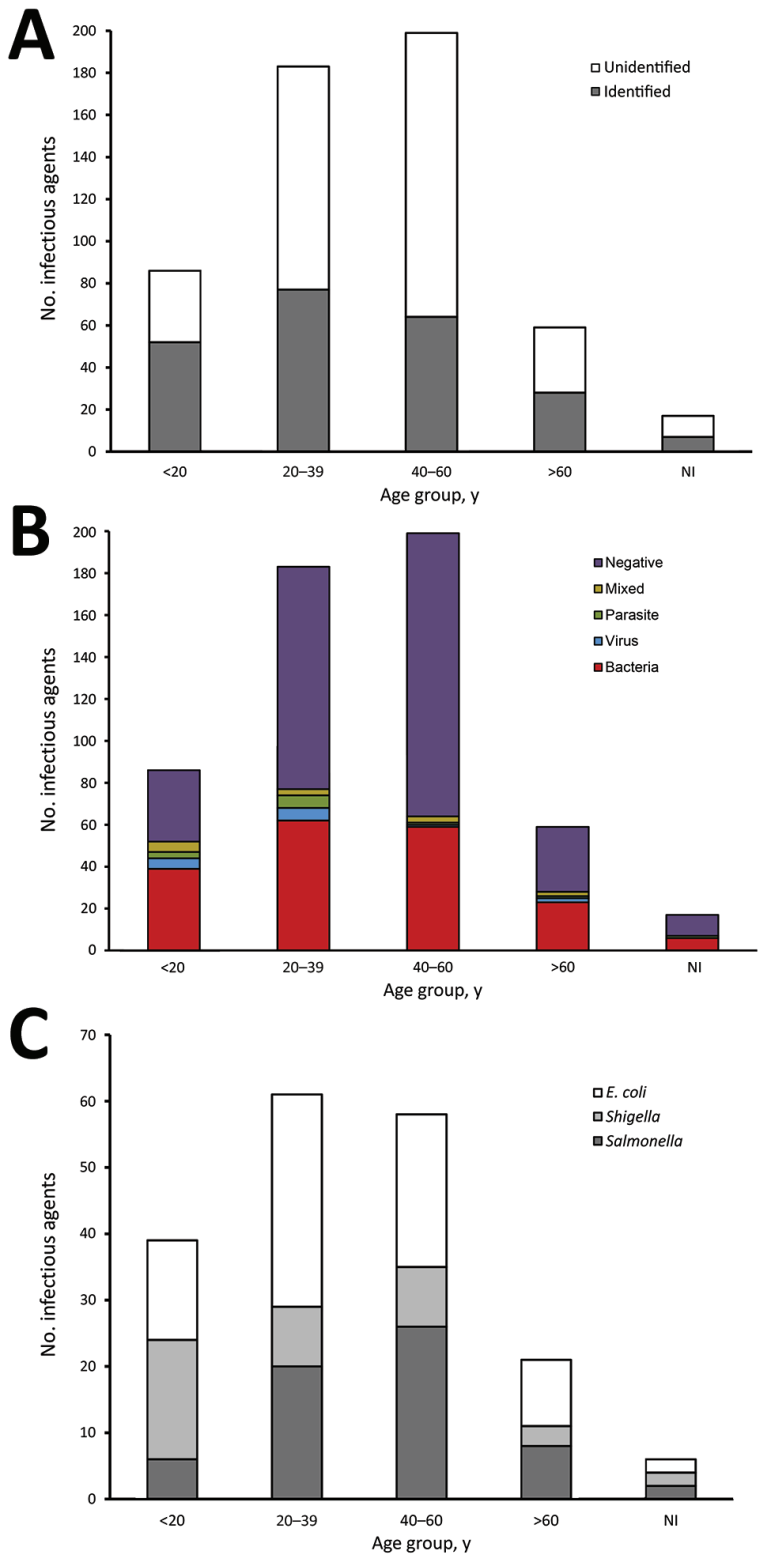
Distribution of infectious agents among persons who acquired enteric infections during their travel for Hajj, 2011–2013, by age group. A) Identified versus unidentified samples; B) type of pathogen; C) bacterial agent. Bacterial agents were the most predominant pathogen detected among all age groups. NI, age not identified.

We also calculated the distribution of the bacterial agents associated with the diarrheal patients during 2011–2013 Hajj seasons by age group (Figure, panel C). *E. coli* was the most frequent species present, detected in 43.39% (n = 82) of the bacteria-positive samples. Of the serovars tested, ETEC was the most common, detected in 25.4% (n = 48) of the positive samples, followed by enteropathogenic *E. coli* (8.47%, n = 16), enteroaggregative *E. coli* (3.7%, n = 7), diffusely adherent *E. coli* (3.7%, n = 7), and enterohemorrhagic *E. coli* (2.12%, n = 4). We detected *Salmonella* spp. in 32.80% (n = 62) and *Shigella*/EIEC in 21.69% (n = 41) of the bacteria-positive samples. We observed significant differences in the distribution of bacterial pathogens across the 3 Hajj seasons (χ^2^ = 12.89; p = 0.01) and among the different age groups (χ^2^ = 21.62; p = 0.01).

### Characterization of Viral and Parasitic Pathogens

We calculated the distribution of the viral and parasitic agents associated with diarrheal infections of pilgrims during the 2011–2013 Hajj seasons (Table 3). Screening for adenoviruses, astroviruses, noroviruses, and rotaviruses showed rotaviruses were most common, detected in 42.86% (n = 6) of the samples positive for the screened viruses. Astroviruses were detected in 21.43% (n = 3), noroviruses in 28.57% (n = 4), and adenoviruses in 7.14% (n = 1) of the virus-positive samples. We used reverse transcription PCR and Sanger sequencing to determine the genotypes of the astroviruses, noroviruses, and rotaviruses detected (Technical Appendix Table 5). All norovirus genotypes identified were recovered from pilgrims from inside Saudi Arabia. Also, 80% of the identified astrovirus genotypes were recovered only from pilgrims from inside Saudi Arabia (astrovirus 2 or 5), whereas the single astrovirus 1 genotype was recovered from a pilgrim from Morocco (Technical Appendix Table 5).

*Giardia* spp. was the most common parasitic agent, identified in 83.33% (n = 10) of the parasite-positive samples, followed by *Cryptosporidium* spp. in 16.66% (n = 2) of the samples. We isolated *Giardia* spp. from patients originating from 10 countries: 4 from Pakistan, 3 from Nigeria, 2 from Bangladesh, and 1 each from Ethiopia, Somalia, Egypt, Jordan, Niger, India, and Afghanistan. We identified *Cryptosporidium* spp. in 2 children (<5 years of age) from Saudi Arabia and 1 older pilgrim (65 years of age) from Chad (Technical Appendix Table 6).

### Relationship between Severity of Diarrheal Disease and Etiologic Agent

We calculated the distribution of the etiologic agents by severity of disease (Table 4). The percentage of samples with identified etiologic agents was significantly higher in patients with severe cases compared with those with mild cases (OR 1.69; p = 0.01). Similarly, the percentage of bacterial agents was significantly higher in patients with severe cases compared with those with mild cases (OR 1.58; p = 0.04). The main bacterial contributors to the severe disease of Hajj-associated diarrheal illness were *Salmonella*, *Shigella*/EIEC, and ETEC.

### Antimicrobial Drug Resistance

We calculated the distribution of β-lactamase genes among the identified bacterial samples (Table 5). *bla*_CTX-M-15_ and *bla*_NDM_ were the most common antimicrobial resistance genes, associated predominantly with *Salmonella* (n = 25/62) and ETEC (n = 16/48). This finding suggests that 40.32% of *Salmonella* infections and 33.33% ETEC infections associated with the Hajj might be resistant to at least some third-generation cephalosporins, and this number might be growing with successive seasons.

**Table 5:** Distribution of β-lactamase genes among the identified bacterial agents among persons who acquired enteric infections during their travel for Hajj, 2011–2013*

Year/bacteria		β-lactamase genes					
		*bla*_CTX-M-15_	*bla*_NDM_	*bla*_KPC_	*bla*_IMP_	*bla*_OXA-48_	*bla*_VIM_
2011							
* Salmonella*		3+2†	2†	0	0	0	0
*Shigella*/EIEC		1+1†	1†	0	0	0	0
ETEC		3+4†	4†	0	0	0	0
EAEC		3	0	0	0	0	0
EHEC		2	0	0	0	0	0
DAEC		0	1	0	0	0	0
2012							
* Salmonella*		6	3	0	0	0	0
*Shigella*/EIEC		4	0	0	0	0	0
ETEC		5+1†	1†	0	0	0	0
EPEC		1	0	0	0	0	0
EHEC		2	0	0	0	0	0
* Yersinia enterocolitica*		2+1†	1†	0	0	0	0
2013							
* Salmonella*		10+1†	1†	0	0	0	0
*Shigella*/EIEC		3	0	0	0	0	0
ETEC		2+1†	1†	0	0	0	0
EPEC		6+1†	1†	0	0	0	0
EAEC		1	0	0	0	0	0

*EAEC, enteroaggregative *Escherichia coli*; EHEC, enterohemorrhagic *E. coli*; EIEC, enteroinvasive *E. coli*; EPEC, enteropathogenic *E. coli*; ETEC, enterotoxigenic *E. coli*; DAEC, diffusely adherent *E. coli*. †Multidrug-resistant; both *bla*_CTX-M-15_ and *bla*_NDM_ were detected.

## Discussion

Enteric infections are commonly associated with mass gathering events, including the annual Hajj pilgrimage to Mecca, Saudi Arabia. The host country and the country of origin of many of the pilgrims are endemic for enteric pathogens and increasingly high levels of antimicrobial resistance. In addition, the lack of effective vaccines against major bacterial infections is challenging (*30*). These circumstances raise serious public health challenges for Saudi Arabia, with potential intercontinental and global implications. A key challenge is the paucity of information available on the structure of the circulating enteric pathogens during Hajj. Comprehensive information on the etiologic agents associated with Hajj-associated diarrheal disease is lacking (*14*). Recent studies have found increased rates of carriage of multidrug-resistant bacteria, including *Salmonella* spp., (*19*) *E. coli* (*20*), and *Acinetobacter baumannii* (*20*) in pilgrims returning home to France after performing Hajj. However, these studies have only focused on colonization by antimicrobial-resistant bacteria in a particular host population.

In this study, we used integrated antigenic and molecular approaches to screen 544 fecal samples from pilgrims with medically attended diarrheal illness for 16 pathogens to identify the etiologic agents responsible for patients seeking care at healthcare facilities during 3 consecutive Hajj seasons. Bacterial pathogens were the most common causes of Hajj-associated diarrheal disease, followed by viruses and parasites, and this pattern was maintained during all 3 seasons.

Our data demonstrate that Hajj-associated diarrheal disease is usually caused by 1 bacterial agent, with ETEC, *Salmonella* spp., and *Shigella*/EIEC being the most common. This association is distinct to the pattern of travelers’ diarrhea observed in travelers from Finland, where multiple bacterial pathogens have been identified in 53% of patients with ongoing diarrhea and 25% of those without symptoms (*31*). However, this observation is not surprising; Hajj-associated diarrheal disease is likely to be different from travelers’ diarrhea because of the different populations involved. Most of Hajj pilgrims originate from intermediate- and high-risk regions for enteric pathogens. In contrast, many travelers’ diarrhea patients are nonimmune persons from developed countries who are naive to many of the enteric pathogens encountered and thus are more highly susceptible to infection when traveling overseas (*32*).

Viruses ranked second and parasites third as the most commonly detected pathogens in patients with Hajj-associated diarrhea. Of note, all of the identified noroviruses and most astroviruses and rotaviruses were recovered from pilgrims from inside Saudi Arabia. The emergent norovirus genotype GII.4 that was first identified in Sydney, Australia, in 2012 and subsequently resulted in global outbreaks had already begun circulating among pilgrims from Saudi Arabia in late October and early November of the 2012 Hajj season. Major causes of diarrhea among children living in Saudi Arabia include rotaviruses (accounting for 6.0% incidence), noroviruses (3.5%), astroviruses (1.9%), and adenoviruses (1.4%) (*33*).

The 3 most commonly identified bacteria in our study (*Salmonella* spp., *Shigella* spp., and *E. coli*) have all been identified by the World Health Organization as being among the top 9 bacteria likely to have a serious impact on global public health (*21*). Of particular concern were the presence of extended-spectrum β-lactamase (ESBL) (primarily *bla*_CTX-M-15_) and carbapenemase (e.g., *bla*_NDM_) genes in ≈40% of *Salmonella* spp. and *E. coli*–positive samples collected.

Recently, travelers’ diarrhea has been shown to be an independent risk factor for contracting ESBL-producing *Enterobacteriaceae* (ESBL-PE) but not carbapenemase-producing *Enterobacteriaceae* (CPE), with the rate of acquisition varying by destination (*34*). Saudi Arabia and the countries of origin for many of the pilgrims are countries at high risk for the acquisition of diarrheal (*9*,*32*) and ESBL-PE infections (*34*). Recent surveillance studies have also reported increasing prevalence of CPE and ESBL-PE isolates in the Gulf Cooperation Council countries (*35*), with some research institutes in Saudi Arabia finding that up to 65% of *E. coli* isolates are ESBL producers (*36*). Recently, the rates of *bla*_CTX-M-15_ infection in Hajj pilgrims have been found to be 31% in 2013 and 34.83% in 2014 (*37*). 

Collectively, these results suggest that further epidemiologic investigations need to be carried out during pilgrimages to identify potential food sources of pilgrim infections. In addition, antimicrobial drug susceptibility testing is needed to inform treatment.

This study used a retrospective approach and 1 anonymized specimen from each patient enrolled in the study. One advantage of this approach is that the study population is more representative of the highly diverse Hajj population, with samples collected from patients originating from 40 different countries. However, a prospective approach with pre- and post-Hajj samples collected from each patient would have provided information on the role of the pilgrimage in contracting the pathogens identified.

In addition, even though integrated molecular and antigenic approaches were used, >50% of the tested samples had no identifiable etiologic agent. These samples require further examination using more comprehensive high-throughput sequencing and metagenomic approaches. High-throughput shotgun sequencing has been used successfully to study population structures and define the epidemiologic links of many enteric pathogens (*38*–*42*). Moreover, metagenomic approaches have been used successfully to identify viral (*43*,*44*) and bacterial (*45*) agents associated with enteric infections. This approach could enable estimation of the ratio of pathogenic to commensal bacteria in pilgrims’ guts, thereby characterizing the acquisition of potential pathogens and their dynamics before and during infections.

Finally, in this study, the assessment of antimicrobial drug susceptibility was only performed by detecting resistance-related genes. The presence of such genes does not necessarily mean the pathogen identified is carrying them, and these genes might be associated with other commensal carriage. We focused on those resistance genes that are posing the most risk to global health and can be easily shared among the *Enterobacteriaceae*, rather than the genes that can confer resistance to the antibiotics widely used for treating enteric infections.

The data we have collected are alarming and highlight the need for further studies to explore the impact of Hajj on public health in Saudi Arabia and globally. Longitudinal studies are required to monitor the changes in colonization patterns of pilgrims during the Hajj, identify the key factors that control these changes, detect the emergence of novel variants (particularly those associated with drug resistance), and understand the dynamics of disease transmission. In addition, active surveillance for enteric diseases is needed to define the potential impact of Hajj on the baseline status of enteric infections in residents of Saudi Arabia and to investigate foodborne outbreaks of disease in a timely manner.

Technical Appendix Description of primers used and additional characteristics of enteric infections associated with travel for Hajj, 2011–2013.
