# Supplementary material for: Enteric Infections Circulating during Hajj Seasons, 2011–2013
Source: Emerg Infect Dis. 2017 Oct;23(10):1640–9. doi: 10.3201/eid2310.161642 (PMC5621540; doi:10.3201/eid2310.161642)
Supplement: Technical Appendix — Description of primers used and additional characteristics of enteric infections associated with travel for Hajj, 2011–2013. [file 16-1642-Techapp-s1.pdf]

Article DOI: https://doi.org/10.3201/eid2310.161642

Enteric Infections Circulating during Hajj Seasons, 2011–2013

Technical Appendix

Technical Appendix Table 1. Details of the primers used in the bacterial characterization

Primer sequence (5′→3′)	Target	PCR amplicon size, bp	Bacterial species
1. GAGCGAAATAATTTATATGTG	VT	518	EHEC
2. TGATGATGGCAATTCAGTAT			
3. CTGAACGGCGATTACGCGAA	*eae*	917	EPEC
4. CGAGACGATACGATCCAG			
5. AATGGTGCTTGCGCTTGCTGC	*bfpA*	326	
6. GCCGCTTTATCCAACCTGGTA			
7. GTATACACAAAAGAAGGAAGC	*aggR*	254	EAEC
8. ACAGAATCGTCAGCATCAGC			
9. GCACACGGAGCTCCTCAGTC	LT	218	ETEC
10. TCCTTCATCCTTTCAATGGCTTT			
11. GCTAAACCAGTAGAG(C)TCTTCAAAA	ST	147	
12. CCCGGTACAG(A)GCAGGATTACAACA			
13. GAACGTTGGTTAATGTGGGGTAA	*daaE*	542	DAEC
14. TATTCACCGGTCGGTTATCAGT			
15. AGCTCAGGCAATGAAACTTTGAC	*virF*	618	*Shigella* spp./EIEC
16. TGGGCTTGATATTCCGATAAGTC			
17. CTCGGCACGTTTTAATAGTCTGG	*ipaH*	933	
18. GTGGAGAGCTGAAGTTTCTCTGC			
19. TATGCCCCATCGTGTAGTCAGAAC	ITS	312	*Salmonella* spp.
20. TGCGGCTGGATCACCTCCTT			
21. GTTAATGCTGTCTTCATTTGGAGC	YST	145	*Yersinia enterocolitica*
22. GACATCCCAATCACTACTGACTTC			
23. AGCAAGAGCATTGTTGTTCCTACC	RTX-A	120	*Vibrio cholerae*
24. ACTTCCCTGTACCGCACTTAGAC			
25. GACTTCGTGCAGATATGGATGCTT	*hipO*	344	*Campylobacter jejuni*
26. GCTATAACTATCCGAAGAAGCCATCA			

Technical Appendix Table 2. Details of the primers used in the viral genotyping

Primer sequence (5′→3′)	Target	PCR amplicon size, bp
1. TCAGATGCATTGTCATTGGT	Astrovirus	449
2. CAACTCAGGAAACAGGGTGT		
3. GGCTTTAAAAGAGAGAATTTCCGTCTGG	Rotavirus G typing	1062
4. GGTCACATCATACAATTCTAATCTAAG		
5. TGGCTTCGCCATTTTATAGACA	Rotavirus P typing	876
6. ATTTCGGACCATTTATAACC		
7. CCAACCCARCCATTTACA	Norovirus GI	330
8. CTGCCCGAATTYGTAAATGA		
9. CCRCCNGCATRHCCRTTRTACAT	Norovirus GII	344
10. CNTGGGAGGGCGATCGCAA		

Technical Appendix Table 3. Primer sequences for *Enterobacteriaceae* resistance testing

Primer sequence (5′→3′)	Target	PCR amplicon size, bp
1. CACACGTGGAATTTAGGGACT	*bla*CTX-M-15	996
2. GCCGTCTAAGGCGATAAACA		
3. CTACCGCAGCAGAGTCTTTGC	*bla*IMP	591
4. GAACAACCAGTTTTGCCTTACC		
5. ATCTGACAACAGGCATGACG	*bla*KPC	452
6. GACGGCCAACACAATAGGTG		
7. GCAGGTTGATCTCCTGCTTG	*bla*NDM	203
8. ACGGTTTGGCGATCTGGT		
9. GCGTGGTTAAGGATGAACAC	*bla*OXA-48	438
10. CATCAAGTTCAACCCAACCG		
11. GATGGTGTTTGGTCGCATA	*bla*VIM	390
12. CGAATGCGCAGCACCAG		

Technical Appendix Table 4. The distribution of cases according to the country of origin

	Year			
Country	2011	2012	2013	Total (%)
Afghanistan	1	16	1	18 (3.31)
Algeria	0	2	0	2 (0.37)
Australia	0	1	0	1 (0.18)
Azerbaijan	0	1	0	1 (0.18)
Bangladesh	5	25	14	44 (8.09)
Benin	0	1	0	1 (0.18)
Burma	0	1	1	2 (0.37)
Canada	0	3	0	3 (0.55)
Chad	0	0	1	1 (0.18)
China	0	1	1	2 (0.37)
Egypt	21	34	15	70 (12.87)
Ethiopia	4	0	0	4 (0.74)
Ghana	0	2	2	4 (0.74)
Guinea	0	1	0	1 (0.18)
India	8	24	2	34 (6.25)
Indonesia	3	2	0	5 (0.92)
Iraq	1	0	0	1 (0.18)
Jordan	3	0	0	3 (0.55)
Kazakhstan	0	1	0	1 (0.18)
Mali	1	1	2	4 (0.74)
Malaysia	1	0	0	1 (0.18)
Morocco	0	5	4	9 (1.65)
Mauritania	0	1	2	3 (0.55)
Nepal	0	0	1	1 (0.18)
Niger	0	1	1	2 (0.37)
Nigeria	1	43	38	82 (15.07)
Oman	1	0	0	1 (0.18)
Pakistan	11	18	6	35 (6.43)
Palestine authority	0	1	0	1 (0.18)
Philippines	0	0	1	1 (0.18)
Saudi Arabia	31	78	26	135 (24.82)
Somalia	1	0	0	1 (0.18)
Sudan	5	1	0	6 (1.1)
Syria	3	2	0	5 (0.92)
Tunisia	0	0	2	2 (0.37)
Turkey	2	2	0	4 (0.74)
United Kingdom	0	3	2	5 (0.92)
Union des Comores	0	1	0	1 (0.18)
United States of America	1	1	0	2 (0.37)
Yemen	11	17	6	34 (6.25)
Country not identified	3	7	1	11 (2.02)
Total samples	118	297	129	544
No. countries	20	30	20	40

Technical Appendix Table 5. Viral genotypes and associated data

Virus	Genotype	Year	Age, y	Sex	Country
Astrovirus					
	HAstV2	2012	32	Male	Saudi Arabia
	HAstV2	2012	9	Female	Saudi Arabia
	HAstV2	2012	36	Female	Saudi Arabia
	HAstV5	2012	2	Male	Saudi Arabia
	HAstV1	2013	56	Male	Morocco
Norovirus					
	GII.3	2011	1	Female	Saudi Arabia
	GI.6	2011	12	Female	Saudi Arabia
	GII.1	2012	33	Female	Saudi Arabia
	GII.4 Sydney	2012	79	Female	Saudi Arabia
Rotavirus					
	G1P[8]	2011	24	Female	Yemen
	G3P[8]	2011	1	Female	Saudi Arabia
	G4P[8]	2011	37	Male	Pakistan
	GxP[8]	2011	30	Male	Saudi Arabia
	G1P[8]	2012	53	Male	USA
	G3P[8]	2012	5	Male	NN
	G1P[8]	2012	1	Male	Saudi Arabia
	GxP[8]	2012	NN	Male	Egypt
Adenovirus					
	ND	2012	39	Male	Egypt
	ND	2012	58	Male	Bangladesh
	ND	2013	3	Male	Pakistan
	ND	2013	0.5	Male	Saudi Arabia

Technical Appendix Table 6. Parasitic agents and associated data*

Parasite	Year	Age, y	Sex	Country
Giardia	2011	30	M	Ethiopia
Giardia	2011	11	F	Somalia
Giardia	2011	31	M	Egypt
Giardia	2011	40	M	Jordan
Giardia	2012	33	M	India
Giardia	2012	68	M	Pakistan
Giardia	2012	10	F	Afghanistan
Giardia	2012	32	M	Nigeria
Giardia	2012	ND	M	Nigeria
Giardia	2012	60	M	Bangladesh
Giardia	2012	35	F	Niger
Giardia	2012	65	M	Pakistan
Giardia	2012	58	M	Bangladesh
Giardia	2012	32	M	Pakistan
Giardia	2013	32	F	Nigeria
Giardia	2013	3	M	Pakistan
Cryptosporidium	2012	3	M	Saudi Arabia
Cryptosporidium	2012	2	F	Saudi Arabia
Cryptosporidium	2013	65	M	Chad

*ND, not determined.

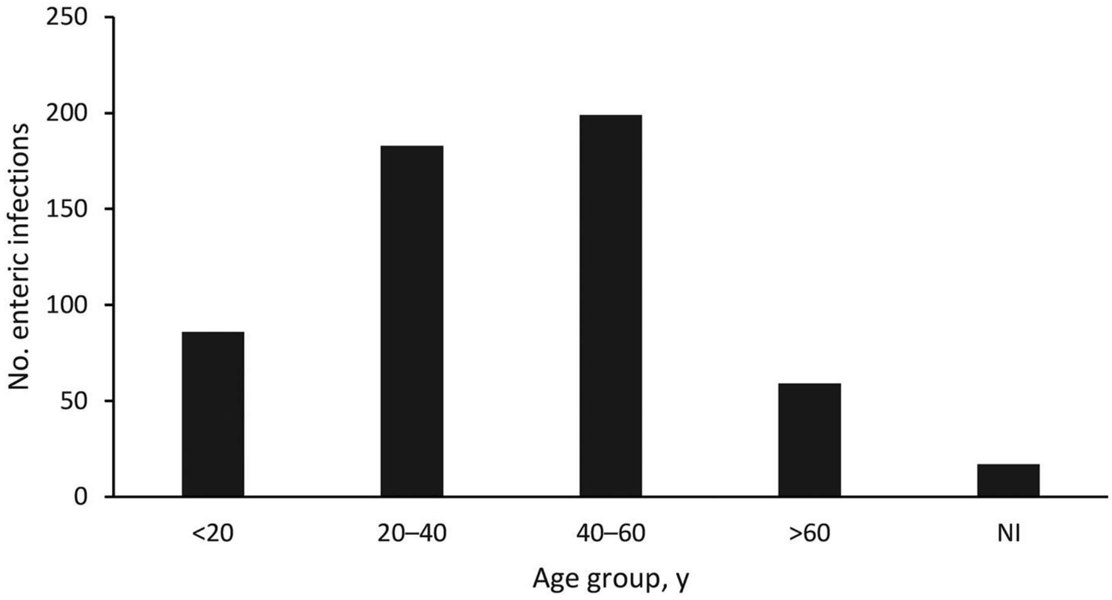

Technical Appendix Figure. Number of persons who had diarrheal symptoms associated with their travel during Hajj seasons, 2011–2013, by age group. NI, age not identified.
